# Surfactin secreted by *Bacillus amyloliquefaciens* Ba01 is required to combat *Streptomyces scabies* causing potato common scab

**DOI:** 10.3389/fpls.2022.998707

**Published:** 2022-11-01

**Authors:** Ru-Ying Feng, Yi-Hsuan Chen, Chih Lin, Chia-Hsin Tsai, Yu-Liang Yang, Ying-Lien Chen

**Affiliations:** ^1^ Master Program for Plant Medicine, National Taiwan University, Taipei, Taiwan; ^2^ Department of Plant Pathology and Microbiology, National Taiwan University, Taipei, Taiwan; ^3^ Plant Pathology Division, Taiwan Agricultural Research Institute, Taichung, Taiwan; ^4^ Agricultural Biotechnology Research Center, Academia Sinica, Taipei, Taiwan

**Keywords:** potato common scab, *Streptomyces scabies*, *Bacillus amyloliquefaciens*, Surfactin, *srf* gene cluster

## Abstract

Potato common scab, which is mainly caused by the bacterium *Streptomyces scabies*, occurs in key potato growing regions worldwide. It causes necrotic or corky symptoms on potato tubers and decreases the economic value of potato. At present, there is no recommended chemical or biological control for combating potato common scab in Taiwan. It can only reduce the occurrence by cultivation control, but the efficacy is limited. Previously we found that *Bacillus amyloliquefaciens* Ba01 could control potato common scab in pot assay and in the field. The potential anti-*S. scabies* mechanism was associated with surfactin secretion, but further molecular dissection was not conducted. Thus, in this study we aimed to determine whether surfactin is the main compound active against *S. scabies* by knocking out the *srf* gene cluster in Ba01. The cloning plasmid pRY1 was transformed to Ba01 by electroporation for in-frame deletion. Two independent Δ*srf* mutants were obtained and confirmed by specific primers and mass spectrometry. The swarming ability and *S. scabies* inhibition was significantly decreased (*P*<0.001) in Δ*srf* mutants. The swarming ability of Δ*srf* mutants could be restored by the addition of surfactin. Furthermore, we found that Ba01 formed wrinkled biofilm in MSgg liquid medium, while Δ*srf* mutants formed biofilm abnormally. Furthermore, the α-amylase, protease and phosphate-solubilizing ability of Δ*srf* mutants was decreased, and the mutants could not inhibit the growth and sporulation of *S. scabies* on potato tuber slices. In conclusion, *srf* gene cluster of *B. amyloliquefaciens* Ba01 is responsible for the secretion of surfactin and inhibition of *S. scabies*.

## Introduction

Potato is one of the four major food crops in the world but is easily infected with multiple diseases including common scab, late blight, bacterial wilt and soft rot. Potato common scab is mainly caused by gram positive bacteria from the *Streptomyces* genus including *S. scabies*, *S*. *acidiscabies*, *S*. *turgidiscabies* and *S. ipomoeae* (Li et al., 2019). The infected potato may exhibit necrotic lesions or corky symptoms on tuber surfaces, thus reducing tuber quality and marketability. Scab symptoms are mainly caused by secreted thaxtomins, such as thaxtomin A, a cellulose biosynthesis inhibitor and key pathogenicity determinant of most *Streptomyces* species (Lawrence et al., 1990; Loria et al., 1995). Although thaxtomin A acts as the main phytotoxin in the development of scab symptom, other phytotoxins can also play significant role in disease development (Li et al., 2019). For instance, Bignell team demonstrated that nigericin and geldanamycin secreted by *Streptomyces* sp. 11-1-2 were phytotoxic to potato tuber tissue (Diaz-Cruz et al., 2022). Currently, development of effective control strategies for potato common scab is challenging due to limited knowledge about the genetic diversity of *S. scabies* and genetic differences in multiple potato cultivars (Lerat et al., 2009). The traditional cultivation control includes crop rotation, irrigation, soil amendment to lower pH, disease-free tuber seed and disease resistant potato cultivars (Dees and Wanner, 2012). Biological control of potato common scab using *Bacillus* spp., *Pseudomonas* spp. and non-pathogenic *Streptomyces* spp. is an emerging area (Arseneault et al., 2016; Lin et al., 2018; Zhang et al., 2020) as their use can reduce environmental pollution produced by chemical pesticides. In our previous study, we found that *B. amyloliquefaciens* Ba01 attenuated the disease severity of potato common scab in pot assays (from 55.6% to 4.2%) and field trial (from 14.4% to 5.6%, achieving 61.1% control effectiveness) *via* secreting surfactin, iturin A and fengycin as potential antagonistic mechanisms (Lin et al., 2018). However, the study did not provide genetic evidence showing that *Bacillus* genes responsible for synthesis of surfactin, iturin A or fengycin are required to combat potato common scab.

*Bacillus* spp. can secrete many secondary metabolites including lipopeptides (surfactin, fengycin and iturin), polyketides (difficidin, bacillaene and macrolactin) and volatiles, which may directly inhibit the growth or sporulation of plant pathogens or elicit induced systemic resistance (ISR) of the plant host (Ongena and Jacques, 2008; Soni and Keharia, 2021). Surfactin or biosurfactant, a lipoheptapeptide, was first isolated in 1968 from *B. subtilis* (Arima et al., 1968), and its synthesis required multiple enzymes called nonribosomal peptide synthetases (Theatre et al., 2021). The surfactin family comprises various peptide moieties of surfactin and thus provides a broad spectrum of biological activity. Surfactin secretion is required for *Bacillus* spp. to control various plant pathogens or diseases. Luo team demonstrated that *srfA* of *B. subtilis* 916 was required for surfactin production and antifungal activity against *Fusarium oxysporum* (Luo et al., 2015). Nifakos team demonstrated that *B. velezensis* Bvel1 could control bunch rot caused by *Botrytis cinerea* in postharvest table grapes, and the potential mechanism was associated with secretion of surfactin and other secondary metabolites, but did not provide evidence on the gene level (Nifakos et al., 2021). Chen team showed that secretion of crude lipopeptides including surfactin, fengycin and iturin by *B. velezensis* FJAT-46737 exhibited antagonistic activity against *Ralstonia solanacearum* causing tomato bacterial wilt (Chen et al., 2020). Sarwar team revealed that surfactin A purified from *Bacillus* NH-100 and NH-217 possessed biocontrol activity against rice bakanae disease (Sarwar et al., 2018). Jin team proved that surfactin produced by *B. velezensis* HN-2 could inhibit the growth of *Xanthomonas oryzae* pv. *oryzae* causing rice disease (Jin et al., 2020). In addition to directly inhibiting the growth of plant pathogens, surfactin secreted from *Bacillus* also has the ability to induce plant defense responses. For example, Cawoy team showed that induced systemic resistance of the host was correlated with the amount of surfactin secreted by *Bacillus* (Cawoy et al., 2014). Wang team demonstrated that surfactin secreted by *B. pumilus* W-7 could inhibit potato late blight *via* induced plant defense responses by increasing the expression of biocontrol genes such as *pod*, *pal* and *cat* (Wang et al., 2020). However, the roles of *Bacillus* genes involved in surfactin biosynthesis and targeting potato common scab remain largely unknown.

In this study, we found that *srf* gene cluster of *B. amyloliquefaciens* Ba01 is required for surfactin biosynthesis, swarming ability, biofilm formation and inhibition ability against *S. scabies*, a bacterium that commonly causes potato common scab.

## Materials and methods

### Strains, chemicals, media and growth conditions

The strains and plasmids used in this study are listed in **Table 1**. *Streptomyces scabies* PS07 was grown on yeast malt extract (YME) solid medium at 28°C. For sporulation, *S. scabies* PS07 was grown on solid *Streptomyces* sporulation medium #1 (SSM1) at 28°C for 14 days in the dark (Lin et al., 2018). Spores were collected with a sterilized cotton swab and 1% phosphate buffered saline (PBS) plus 0.1% Tween 20 solution and washed twice with ddH_2_O. Spore suspension was filtered through miracloth (22-25 m**;** Calbiochem, Billerica, MA, USA), while spore concentration was determined by serial dilution. *Bacillus amyloliquefaciens* Ba01 and *Escherichia coli* strains were cultured in Luria-Bertani (LB) broth or on LB solid plates at 37°C.

**Table 1:** Strains and plasmids used in this study.

Strain/plasmid	Description	Source
**Strain**		
*Bacillus amyloliquefaciens*		
Ba01	Wild type	(Lin et al., 2018)
FRY1	Δ*srf* #1	This study
FRY3	Δ*srf* #3	This study
*Streptomyces scabies*		
PS07	Wild type	(Lin et al., 2017)
**Plasmid**		
pMiniMad2	*E. coli*−*Bacillus* shuttle vector, Ap^r^, Em^r^	(Patrick and Kearns, 2008)
pRY1	pMiniMad2 carrying 5′ and 3′ NCR of Ba01 *srf* gene cluster	This study

Media and chemicals used in this study are described below. YME medium (0.4% yeast extract, 1% malt extract, 0.4% dextrose and 2% agar), SSM1 (0.1% MgSO_4_·7H_2_O, 0.05% K_2_HPO_4_, 0.1% yeast extract, 1% soluble starch, 0.1% casein and 2% agar), LB broth (0.5% yeast extract, 1% tryptone and 1% NaCl)/solid medium (1.5% agar), surfactin (dissolved in methanol; Sigma SI-S3523, St. Louis, MO, USA). When necessary, antibiotics were added at the following concentrations: 1 μg/ml erythromycin (Bio Basic, Markham, ON, Canada) plus 25 μg/ml lincomycin (Bio Basic, Markham, ON, Canada) (*mls* for *Bacillus* spp.) (Tsui et al., 2004; Patrick and Kearns, 2008), 100 μg/ml ampicillin (MDBio, New Taipei City, Taiwan) (for *E. coli*). Minimal salts glutamate glycerol (MSgg) medium (0.026 g KH_2_PO_4_, 0.061 g K_2_HPO_4_, 2.09 g MOPS and 0.04 g MgCl_2_·6H_2_O per 100 ml of dH_2_O, and adjust pH to 7.0 using KOH)/solid (1.5% agar). The MSgg base was autoclaved, cooled down to room temperature and supplemented with filtered (0.22 μm) 0.1 ml of 0.7 M CaCl_2_, 0.1 ml of 100 mM MnCl_2_, 0.1 ml of 50 mM FeCl_3_, 0.1 ml of 1 mM ZnCl_2_, 0.1 ml of 2 mM thiamine, 0.57 ml of 86% glycerol and 10 ml of 5% K-glutamate.

### Plasmid construction and transformation

**Figure 1:**
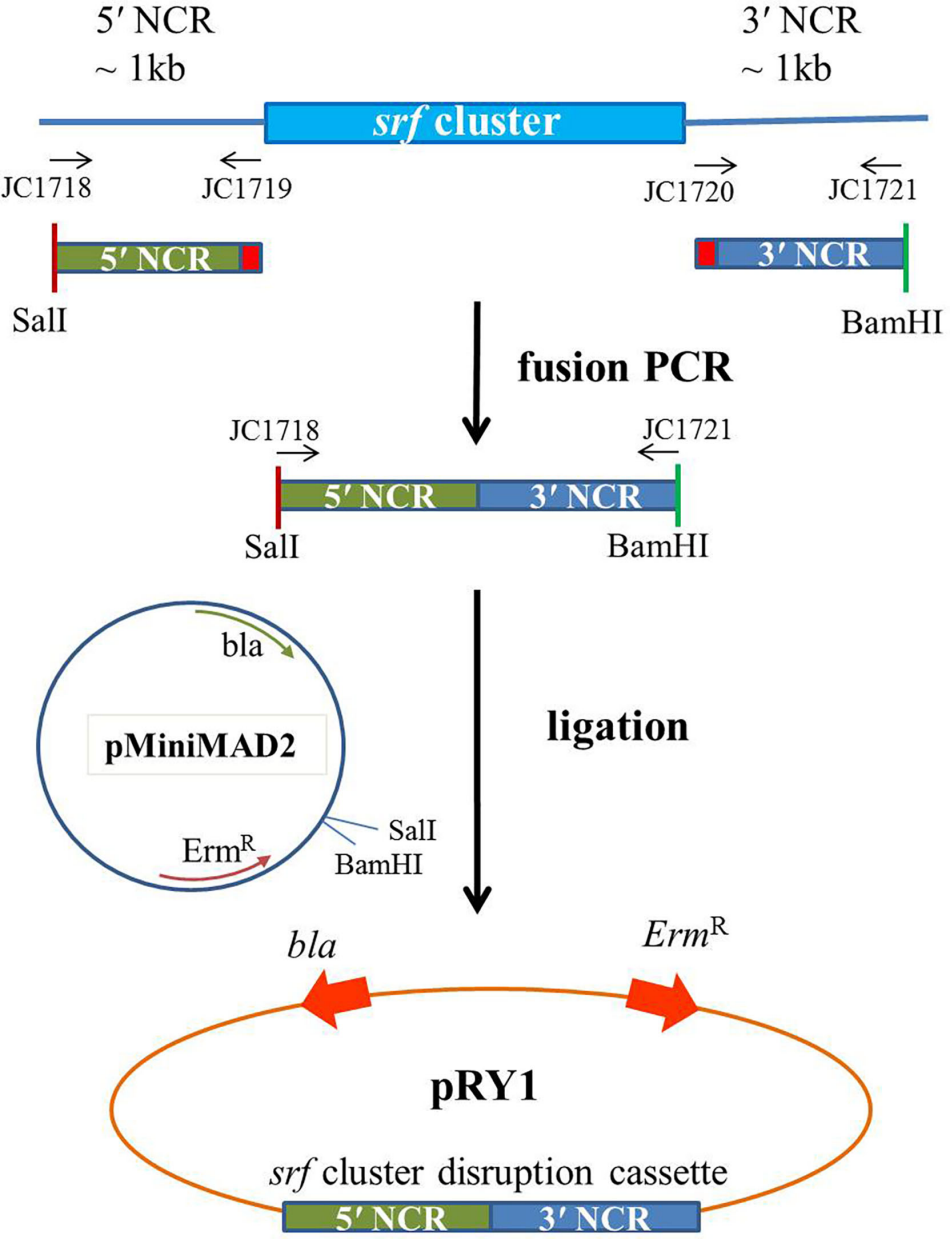
Scheme of the plasmid construction for making Δ*srf* mutant in *B amyloliquefaciens* Ba01. Primers JC1718/1719 and JC1720/1721 were used to amplify 5′ and 3′ NCR sequences of *srf* gene cluster of *B amyloliquefaciens* Ba01, respectively, and fused together by fusion PCR using primers JC1718/1721. After enzyme digestions the ~2 kb cassette was ligated to pMiniMAD2 in order to generate disruption plasmid pRY1.

The 5′ and 3′ non-coding region (NCR) fragments (~1 kb) of *srf* gene cluster of Ba01 were amplified using primers JC1718/1719 and JC1720/1721, respectively. The primers JC1719/17120 have 27 bp complementary sequence for fusion PCR (Gao et al., 2017). These two fragments were fused together by fusion PCR using primers JC1718/1721, and digested with BamHI and SalI. Then the fragment was ligated into the BamHI and SalI cutted pMiniMAD2, which carried a temperature-sensitive origin of replication and an erythromycin resistance cassette to generate plasmid pRY1 (**Table 1**; **Figure 1**). Plasmid pRY1 was transformed into Ba01 using a high osmolarity electroporation method with slight modifications (Zhang et al., 2011; Lu et al., 2012). In brief, single colony of Ba01 was grown in 3 ml LB broth overnight at 37°C with shaking at 200 rpm. Culture was 16-fold diluted with LB broth containing 0.5 M sorbitol, then incubated for about 2.5 h at 37°C with shaking in order to achieve optical density at 600 nm (OD_600_) 0.85 to 0.95. The culture was cooled on ice for 10 min and harvested by centrifugation for 10 min with 8,000 g at 4°C. Then, the cells were washed four times with ice-cold electroporation medium (ETM) (0.5 M sorbitol, 0.5 M mannitol and 10% glycerol), and electro-competent cells were resuspended in 1/40 (v/v) ETM (~2×10^10^ CFU/ml). For electroporation, 100 μl of Ba01 competent cells were mixed with 1 to 3 μg plasmid pRY1, and transferred into an ice-cold electroporation cuvette (Bio-Rad #1652086, Hercules, CA, USA). After incubation on ice for 1 min, the mixture was shocked by MicroPulser Electroporator (Bio-Rad #165-2100, Hercules, CA, USA) at 2.1 kV/cm for 4.5 to 5.0 ms. Then 1 ml of recovery medium (LB broth containing 0.5 M sorbitol and 0.38 M mannitol) was added to the mixture and incubated at 37°C for 3 h with agitation. After incubation, the culture was spread on *mls* agar plate (LB agar plate containing 1 μg/ml erythromycin and 25 μg/ml lincomycin) at 28°C for selecting transformants. Transformants were initially checked by colony PCR with primers JC1512/1513 by amplifying Erm^R^ cassette on plasmid pRY1.

### In-frame deletion of the *srf* gene cluster in *B. amyloliquefaciens* Ba01

**Figure 2:**
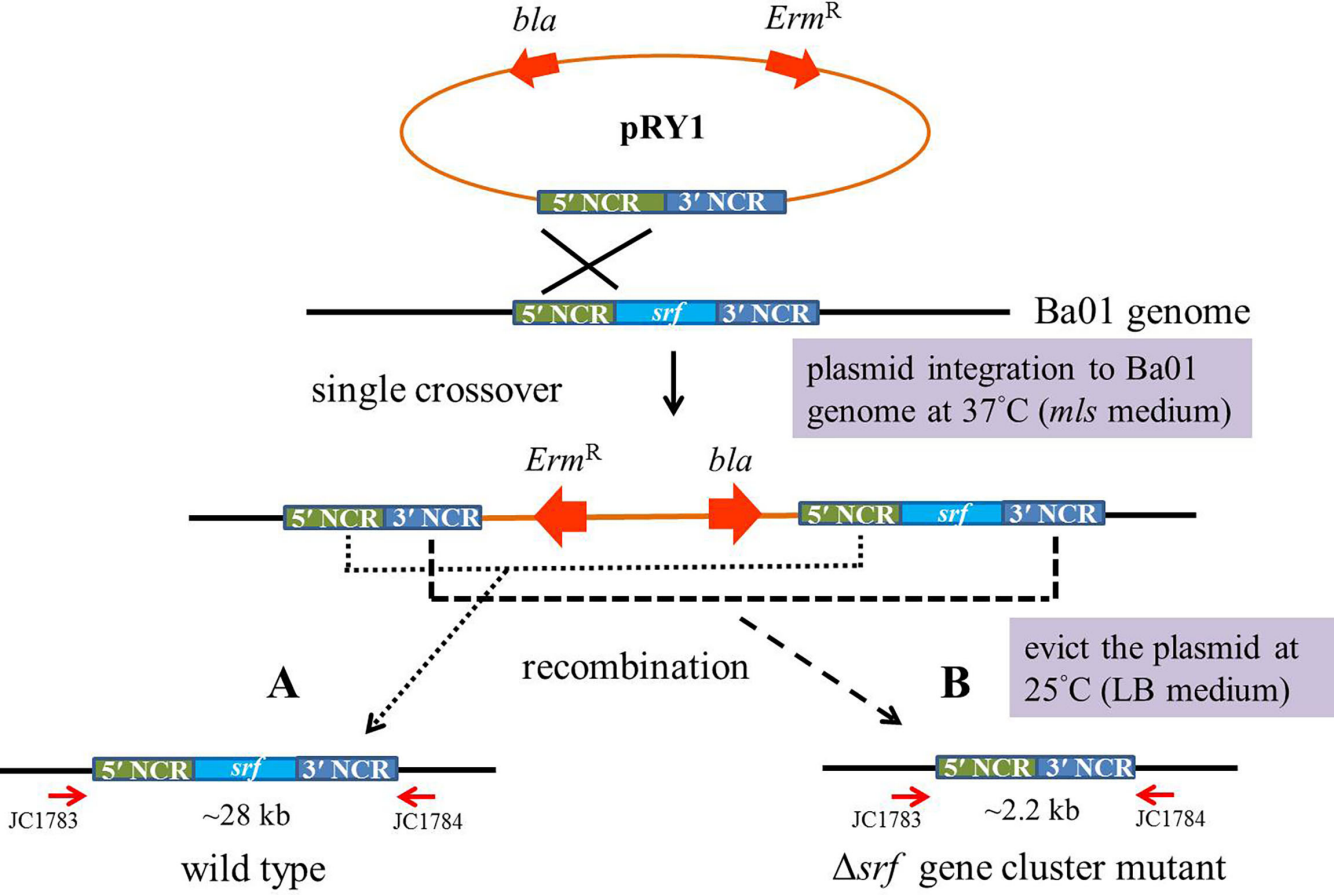
Scheme of in-frame markerless deletion method to construct the *srf* gene cluster mutants in *B amyloliquefaciens* Ba01. Single crossover recombinants were obtained by culturing the transformants at restrictive temperature (37°C) for plasmid pRY1 integration into Ba01 genome and using *mls* medium for selection. To evict the plasmid from Ba01 genome after homologous recombination, the strain was incubated overnight in 3 ml of LB broth at a permissive temperature (25°C) for plasmid replication. The cell cultures were diluted and plated on LB agar at 37°C for 24 h The colonies were patched on *mls* medium to select *mls*-sensitive colonies. The *srf* gene cluster mutants were checked by PCR with primers JC1783/1784.

Transformants with the erythromycin resistance gene were incubated overnight in 3 ml *mls* media at 28°C with agitation. The cultures were diluted and plated on *mls* solid media for 1 day at 37°C, a restrictive temperature for plasmid integration into the Ba01 genome in order to obtain single crossover recombinants. Potential recombinants were picked and subcultured in 3 ml LB broth overnight at 25°C, then subcultured overnight in fresh LB broth at 1:100 ratio at 25°C, a permissive temperature for plasmid replication. The above procedures were repeated in order to induce second crossover in recombinants. The cultures of potential recombinants were diluted and plated on LB agar plates at 37°C for 1 day, then the colonies were patched on *mls* solid medium to identify *mls*-sensitive colonies (**Figure 2**). Two independent *srf* gene cluster mutants (FRY1 and FRY3) were obtained and checked by PCR with primers JC1468/1469 (for amplification of *srfAD* ORF) and JC1783/1784 (for confirmation of correct integration).

### Cell growth and endospore formation

For measuring cell growth, wild type and Δ*srf* mutants were incubated overnight in 5 ml LB broth at 37°C and diluted to OD_600_ = 1. Then 200 μl of culture was inoculated in 20 ml fresh LB broth at 37°C with agitation. For growth curve experiments, bacterial numbers were calculated by measuring OD_600_ and serial dilution methods after 0, 3, 6, 9, 12, 24 and 30 h. Each experiment was performed in triplicate.

For measuring endospore formation, cultures of the wild type and Δ*srf* mutants were serial diluted and plated on LB agar plates to calculate total bacterial numbers after incubating for 1, 3, 5 and 7 days at 37°C with agitation. Cultures were then incubated at 80°C for 10 min to kill vegetative cells, serial diluted and incubated on LB agar plates at 37°C for 24 h. The ratio of endospore and total bacterial numbers was represented as percentage of endospore production. Each experiment was performed as three replicates. Endospore staining was performed based on Reynolds team with minor modifications (Reynolds et al., 2009). In brief, 20 μl culture of wild type or Δ*srf* mutant were placed on a slide and heat fixed by flame, then the smear was flooded with malachite green, and steamed 5 to 10 min for staining. Then the slides were washed with ddH_2_O, the smear was flooded with safranin and left for 1 min before washing the slide with ddH_2_O in order to remove the secondary stain. The endospores were observed under optical microscope with 100× magnification.

### Disk diffusion assays

One hundred microliters of 10^7^ CFU/ml *S. scabies* spores were spread on YME agar plates, and 6 mm disks were placed on the plates. Then 3 μl culture of wild type or Δ*srf* mutant (OD_600_ = 1) was dropped on the disk, and the plates were incubated at 28°C for 2 days in the dark. The size of the inhibition zone was calculated as below: (diameter of the inhibition zone – diameter of the colony size)/2 (mm). Each experiment contained four replicates.

### MALDI-TOF mass spectrometry analysis

The wild type and Δ*srf* mutants were incubated in 10 ml LB broth with agitation at 37°C for 36 h, and then centrifuged at 13,000 rpm for 5 min to collect the supernatant, which was further extracted twice with ethyl acetate, and finally dissolved in methanol for mass analysis (Han et al., 2005; Liao et al., 2016). One microliter of the sample and surfactin standard (2 mg/ml) were dropped into a sample well. The data were collected using the MALDI TOF/TOF spectrometer (Bruker Autoflex Speed, Agricultural Biotechnology Research Center, Academia Sinica) and analyzed by Bruker Compass version 1.2 software suite.

### Swarming motility

The wild type and Δ*srf* mutants were incubated overnight in LB broth with agitation at 37°C, washed with ddH_2_O and diluted to OD_600_ = 1. Then 5 μl of each culture was spotted on the center of 0.25%, 0.5% or 0.7% solid LB agar plates, and incubated at 37°C for 48 h (Gao et al., 2017). In order to investigate whether surfactin could rescue the swarming motility of Δ*srf* mutants, 10 μl solution containing 1 μg, 20 μg or 100 μg of commercial surfactin (dissolved with methanol) was added on the center of 0.7% solid LB agar plate before adding 5 μl culture of Δ*srf* mutants (OD_600_ = 1) onto the top. The plates were incubated at 37°C for 48 h and the diameter of the colony was measured. Each experiment was performed with five replicates.

### Biofilm formation and colony architecture

For observation of biofilm formation, 10 μl of strains (OD_600 _= 1) were added to 1 ml MSgg broth and incubated in 24-well polystyrene plate at 30°C for 2 days without agitation. Twenty micromolar commercial surfactin was further added into the wells in order to investigate whether surfactin could rescue biofilm formation of Δ*srf* mutants. For colony architecture, 3 μl of strains (OD_600_ = 1) were spotted on the center of MSgg solid medium, and incubated at 30°C for 2 or 4 days. To determine whether commercial surfactin could rescue the colony architecture of Δ*srf* mutants, 5 μl of solution containing 10 μg commercial surfactin was spotted on the center of MSgg solid medium before adding 3 μl of Δ*srf* mutants (OD_600_ = 1).

### Extracellular enzyme activity and phosphate-solubilizing ability

*Bacillus* spp. has the ability to secrete extracellular hydrolases and possesses phosphate-solubilizing ability to defend against pathogens or help plants to absorb nutrients. To investigate whether hydrolases and phosphate-solubilizing ability of Δ*srf* mutants would be affected, the following assays were performed. (i) protease activity assay: 3 μl of strains (OD_600_ = 1) were inoculated on skimmed milk agar (1% peptone and 1.5% agar mixed with 1% skimmed milk) and incubated at 37°C for 48 h. The colorless zone represented colonies with protease-producing ability. (ii) α-amylase activity assay: 3 μl of strains (OD_600_ = 1) were inoculated on the medium (0.2% soluble starch, 1.5% agar, pH = 7) and incubated at 37°C for 48 h, then the plates were stained with iodine solution (0.67% potassium iodide, 0.33% iodine). The colorless zone represented the colony with α-amylase-producing ability. (iii) cellulase activity assay: 3 μl of strains (OD_600_ = 1) were inoculated on carboxymethyl cellulose (CMC) agar medium (1% peptone, 1% yeast extract, 1% carboxymethyl cellulose, 0.5% NaCl, 0.1% KH_2_PO_4_, 1.5% agar, pH = 7) and incubated at 37°C for 48 h, then the colonies were washed with a sterilized cotton swab and ddH_2_O. Plates were then flooded with 0.1% Congo red for 1 h and destained with 1 M NaCl solution for 1 h. The colorless zone represented colonies with cellulase-producing ability. (iv) phosphate-solubilizing ability assay: 3 μl of strains (OD_600_ = 1) were inoculated on Pikovskaya’s agar medium (Pikovskaya, 1948), and incubated at 37°C for 5 days, then the colonies were washed with a sterilized cotton swab and ddH_2_O. The colorless zone represented colonies with phosphate-solubilizing ability.

### Tuber slice assays

Commercial G4 potato tubers of the cultivar Kennebec were used. The surfaces of potato tubers were sterilized with 400 ppm hypochlorous acid water. Cores (1.2 cm diameter) of pith tissue were removed from tubers using a puncher and sliced into pieces 0.25 cm thick. Then 25 μl (10^8^ CFU/ml) of *S. scabies* PS07 spores were inoculated onto potato tuber slices, which were placed on moist filter paper in Petri dishes. After incubating the tuber slices at 28°C for 1 day in the dark, 20 μl of wild type, Δ*srf* mutants (OD_600_ = 1), commercial surfactin (0.1 mg/ml) or ddH_2_O were dropped onto the potato tuber slices. The samples were further incubated in a moist chamber in the dark at 28°C for 5 days before being photographed.

## Results

### *srf* gene cluster was deleted in *B. amyloliquefaciens* Ba01

**Figure 3:**
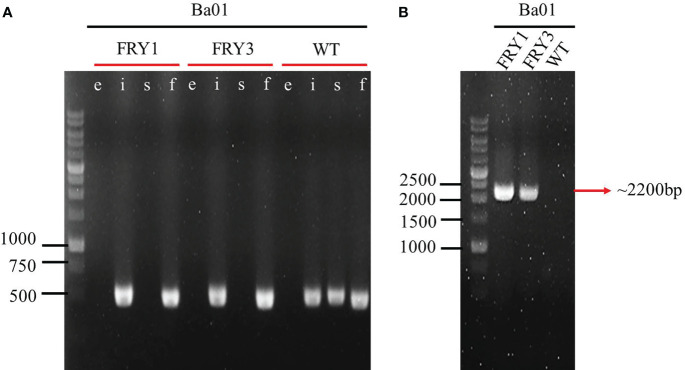
Confirmation of *srf* gene cluster mutants by PCR amplification. **(A)** lane e: *Erm* ^R^ marker (~1100 bp; JC1512/1513), lane i: *ituD* ORF (469 bp; JC1466/1467), lane s: *srfAD* ORF (485 bp; JC1468/1469), lane f: *fenA* ORF (455 bp; JC1470/1471). **(B)** Outside primer of 5′ and 3′ NCR of *srf* gene cluster (~2200 bp; JC1783/1784).

The plasmid pRY1 was constructed and transformed into *B. amyloliquefaciens* Ba01 by electroporation (**Figure 1**). An in-frame markerless deletion method was used to generate Δ*srf* gene cluster mutants (Δ*srf* mutant; **Figure 2**). Two independent Δ*srf* mutants FRY1 and FRY3 were obtained and confirmed by PCR with primer pairs JC1512/1513, JC1466/1467, JC1468/1469 and JC1470/1471 (**Table 2**). In **Figure 3A**, *srfAD* ORF could only be amplified with primers JC1468/1469 in the wild type, but not in the Δ*srf* mutants. Two Δ*srf* mutants were further validated by primers JC1783/1784 (SrfOUT-F/R) in order to verify the correct integration in the genome. In **Figure 3B**, *srf* gene cluster of the wild type was too long (~26 kb) to amplify, while two Δ*srf* mutants could amplify a band of 2.2 kb, a fragment containing only 5′ and 3′ NCR of *srf* gene cluster.

**Table 2:** Primers used in this study.

Primer	Use	Sequence (5′ → 3′)^*^
JC1466	*ituD* ORF	aaaAGTGTATGCCGCACCCTTTT
JC1467	*ituD* ORF	aaaGAGCGATGCGATCTCCTTGG
JC1468	*srfAD* ORF	aaaCCGCCGTTGAGGATTTTGAA
JC1469	*srfAD* ORF	aaaCATGTGGCCGTCCGAAAACT
JC1470	*fenA* ORF	aaaGCGAGAGGCTGGTATTGCAT
JC1471	*fenA* ORF	aaaGAACACCTTTCACTGGCGGA
JC1512	Erm^R^ cassette-F	TAGGCACACGAAAAACAAGTTAAGG
JC1513	Erm^R^ cassette-R	AACCGTGTGCTCTACGACC
JC1718	SrfUP‐F (SalI)	GCC**GTCGAC**ATGGGAATAACTTTTTATCC
JC1719	SrfUP‐R	GGCATCGATATTGCTCCAGAGATACTGTAAAC
JC1720	SrfDN‐F	CAGTATCTCTGGAGCAATATCGATGCCGATCG
JC1721	SrfDN‐R (BamHI)	CGC**GGATCC**ATCTTTAACCATTAAAGGAAAAG
JC1783	SrfOUT-F	GGAGGCTGTTTCTAAGGAAGAATTGAC
JC1784	SrfOUT-R	GACGTTTTATTTTGCCGGTCTGTTG

^*^Bold letters represent restriction sites used for the cloning; underlined letters represent overlapping PCR sequences.

### *srf* gene cluster is required for secreting surfactin in *B. amyloliquefaciens* Ba01

**Figure 4:**
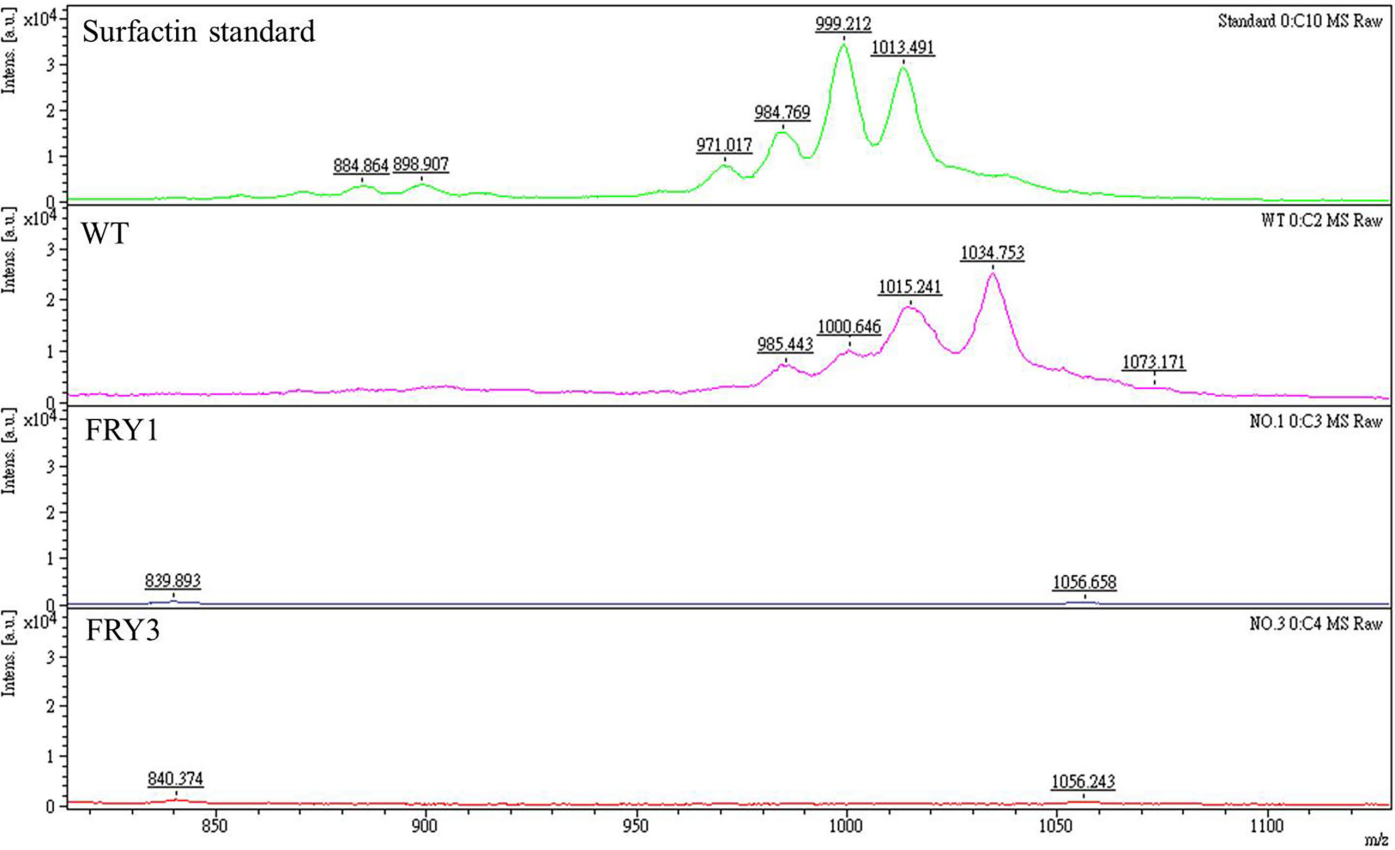
The Δ*srf* mutants lose the ability to secrete surfactins. The supernatants of cultures were collected and extracted twice with ethyl acetate, followed by resuspension with methanol before conducting mass spectrometry analysis. The surfactin peaks were found in the standard and wild type (WT), but deficient in Δ*srf* mutants (FRY1 and FRY3).

Based on mass spectrometry analysis, the peaks of surfactin were detected in the wild type and surfactin standard, but absent in Δ*srf* mutants (**Figure 4**). These results indicated that *srf* gene cluster is essential for secreting surfactin in *B. amyloliquefaciens* Ba01. Besides, we found that the mass-to-charge (*m/z*) ratio of the surfactin from Ba01 was slightly different from the surfactin standard. We speculated that the difference might be because the surfactin standard purchased was extracted from *B. subtilis*, a relative of *B. amyloliquefaciens*.

### The growth kinetics of *B. amyloliquefaciens* Δ*srf* mutants were slightly decreased

**Figure 5:**
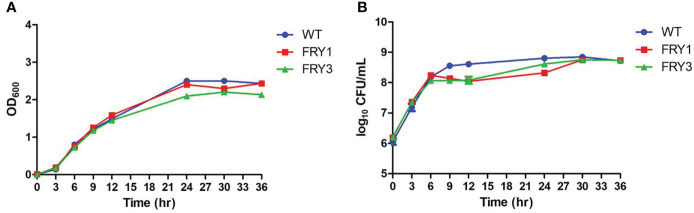
The growth kinetics of Δ*srf* mutants were slightly decreased. **(A)** Growth curves were determined based on the OD_600_ values. **(B)** Growth curves were determined based on the viable cell counts (CFU/ml). The strains were 10-fold diluted and spread on LB agar plates. Total colony numbers were counted after 1 day growth at 37°C.

*B. amyloliquefaciens* Ba01 wild type and Δ*srf* mutants were cultured in LB broth, OD_600_ values were measured by spectrophotometer and bacterial number (colony-forming units) was determined by serial dilution at 0, 3, 6, 9, 12, 24 and 30 h. Although there were no differences between the wild type and Δ*srf* mutants based on OD_600_ values (**Figure 5A**), the colony-forming units of two Δ*srf* mutants were slightly decreased at 9 and 12 h compared with the wild type (*P* < 0.001, *t*-test) (**Figure 5B**).

### The endospore formation was decreased in Δ*srf* mutants of *B. amyloliquefaciens* Ba01

**Figure 6:**
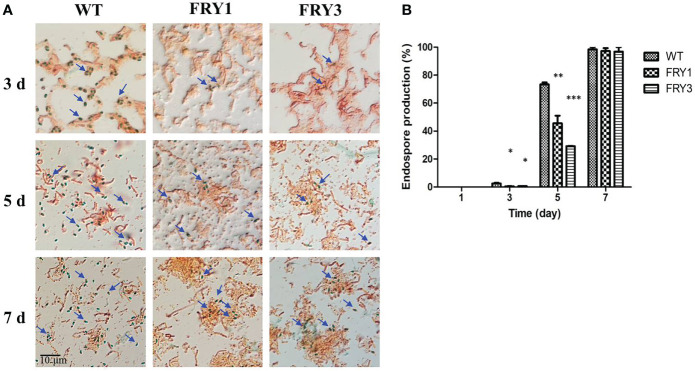
The endospore formation of Δ*srf* mutants was decreased. **(A)** Endospores were stained and observed under a microscope (100×). **(B)** Graph of endospore formation. The strains were treated at 80°C for 10 min to kill vegetative cells and plated on LB agar to calculate the number of endospores. Endospore formation was the ratio of endospore numbers divided by total colony numbers. P values were calculated by *t*-test, “*”, “**” and “***” represent *P* < 0.05, *P* < 0.01 and *P* < 0.001 respectively as compared to the WT.

In the endospore formation test, *B. amyloliquefaciens* Ba01 wild type and Δ*srf* mutants did not form endospores after incubation for 1 day in LB broth. After 3 days, the endospore formation rate of the wild type was 2.5 ± 0.46%, while those of Δ*srf* mutants were significantly reduced (P<0.01) to 0.5 ± 0.09% (FRY1) and 0.7 ± 0.7% (FRY3) (**Figures 6A, B**). After 5 days, the endospore formation rate of the wild type was 73.4 ± 1.35%, while those of Δ*srf* mutants were also significantly reduced (P<0.01) to 45.6 ± 5.49% (FRY1) and 29.1 ± 0.13% (FRY3). Under a microscope, more endospores were observed in the wild type than in the Δ*srf* mutants after incubation for 3 or 5 days (**Figure 6A**). After 7 days, the endospore formation rate of the wild type was 98.3 ± 1.67%, while those of the Δ*srf* mutants were 97.2 ± 2.14% (FRY1) and 96.8 ± 3.2% (FRY3).

### The Δ*srf* mutants showed reduced inhibition activity against *S. scabies*

**Figure 7:**
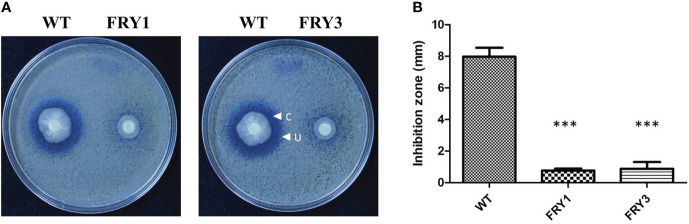
The Δ*srf* mutants reduced the inhibition activity against *S. scabies*. **(A)** Disk diffusion assay. The 10^6^ spores of *S. scabies* PS07 were spread on YME agar plates, and a disk containing 3 μl WT or Δ*srf* mutant culture (OD_600_ = 1) was put on the surface of plates. The plates were incubated at 28°C for 2 days. “C” represents clear inhibition zone, and “U” represents undifferentiated zone. **(B)** Graph of **(A)**. P values were calculated by *t*-test, “***” represents *P* < 0.001 as compared to the WT.

In disk diffusion assays, *B. amyloliquefaciens* Ba01 wild type could effectively inhibit the growth of *S. scabies* PS07 and formed clear inhibition/undifferentiating zones after incubation for 2 days, while Δ*srf* mutants could only form an undifferentiating but not a clear inhibition zone (**Figure 7A**). The clear and undifferentiating inhibition zones produced by the wild type were 4.63 ± 0.49 mm and 7.97 ± 0.57 mm, respectively, while undifferentiating zones produced by Δ*srf* mutants were 0.77 ± 0.12 mm (FRY1) and 0.88 ± 0.42 mm (FRY3), showing significantly reduced (P<0.001) as compared to the wild type (**Figure 7B**).

### Swarming motility was attenuated in Δ*srf* mutants of *B. amyloliquefaciens* Ba01

**Figure 8:**
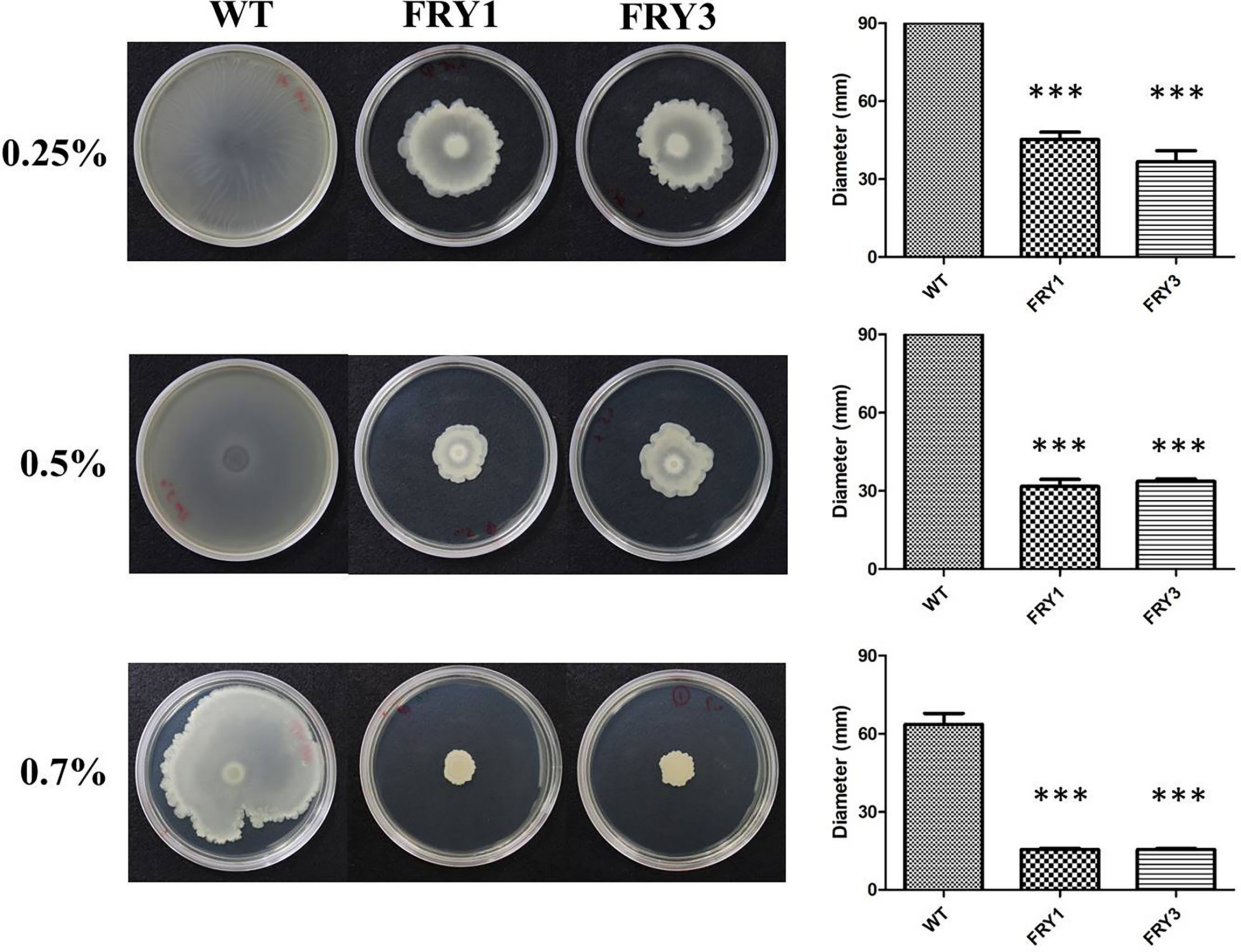
The Δ*srf* mutants showed attenuated swarming motility. Five microliters of strains (OD_600_ = 1) were inoculated on 0.25%, 0.5% or 0.7% solid LB agar at 37°C for 48 h. P values were calculated by *t*-test, “***” represents *P* < 0.001 as compared to the WT.

The wild type could swarm over the 0.25% and 0.5% LB agar plates, while the swarming motility of Δ*srf* mutants was reduced to 45.2 ± 2.8 mm (FRY1) and 36.6 ± 4.31 mm (FRY3) on 0.25% LB agar plates, and 31.7 ± 2.68 mm (FRY1) and 33.7 ± 0.89 mm (FRY3) on 0.5% LB agar plates (**Figure 8**). In addition, the wild type and Δ*srf* mutants swarmed 63.6 ± 4.19 mm, 15.5 ± 0.46 mm (FRY1) and 15.5 ± 0.48 mm (FRY3), respectively, on 0.7% LB agar plates. Overall, the swarming motility of Δ*srf* mutants on 0.25%, 0.5% and 0.7% LB agar plates were significantly decreased compared with the wild type (*P* < 0.001, **Figure 8**).

### Surfactin could rescue the swarming motility of Δ*srf* mutant in *B. amyloliquefaciens* Ba01

**Figure 9:**
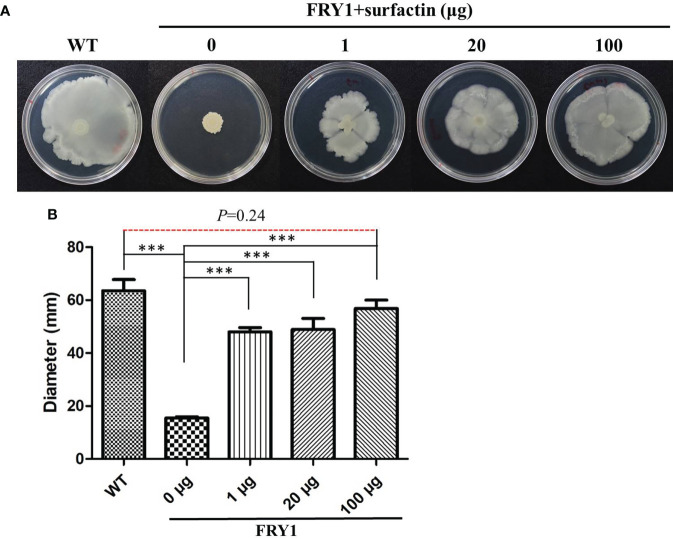
Surfactin could rescue the swarming motility of Δ*srf* mutant. **(A)** Solution of 10 μl containing 1 μg, 20 μg or 100 μg of surfactin were spotted on the center of 0.7% solid LB medium before dropping 5 μl of strains (OD_600_ = 1) onto the top, and then incubated at 37°C for 48 h **(B)** Graph of **(A)**. P values were calculated by *t*-test, “***” represents *P* < 0.001 as compared to the Δ*srf* mutant (FRY1) in the absence of surfactin.

In order to confirm that the defect of swarming motility in Δ*srf* mutants was associated with surfactin acquirement, a surfactin supplementing experiment was conducted. After adding 1, 20 or 100 μg of commercial surfactin on 0.7% LB agar plates, the swarming motility of Δ*srf* mutant (FRY1) was significantly increased compared to Δ*srf* mutant without adding surfactin (**Figures 9A, B**, *P* < 0.001). In addition, there was no difference in the swarming motility between the wild type and Δ*srf* mutant supplemented with 100 μg surfactin (**Figures 9A, B**), indicating that 100 μg of surfactin was sufficient to restore the swarming motility of Δ*srf* mutant to similar to the wild type.

### Biofilm formation was impaired in Δ*srf* mutants of *B. amyloliquefaciens* Ba01

**Figure 10:**
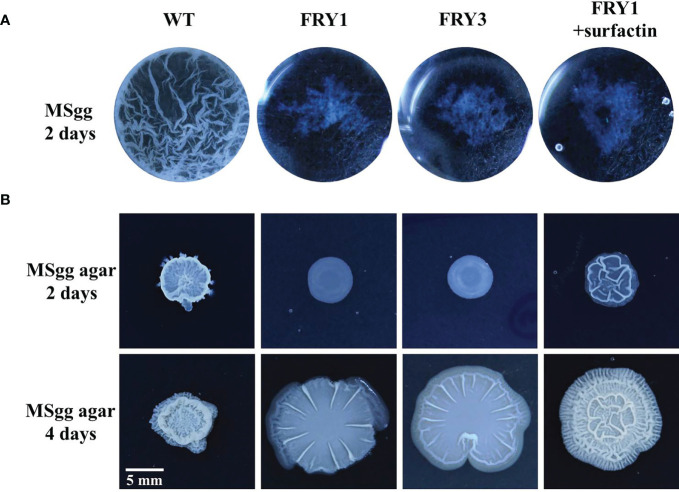
The Δ*srf* mutants lost the ability to form biofilm. **(A)** WT formed a thick and wrinkled biofilm, while Δ*srf* mutants did not. Surfactin did not rescue the biofilm formation of Δ*srf* mutant in liquid MSgg medium. For biofilm formation, 10 μl of strains (OD_600_ = 1) were added to 1 ml MSgg broth and incubated in a 24-well plate at 30°C for 2 days. The addition of 20 μM of surfactin was to determine if surfactin could rescue biofilm formation. **(B)** For colony morphology, 3 μl of strains (OD_600_ = 1) were spotted on the center of MSgg solid medium, and then incubated at 30°C for 2 or 4 days. To determine if surfactin could rescue colony morphology, solution of 5 μl containing 10 μg of surfactin was spotted on the center of MSgg solid medium before adding 3 μl of FRY1 (OD_600_ = 1).

The wild type could form thick and wrinkled biofilm in liquid MSgg medium after incubation for 2 days, while Δ*srf* mutants exhibited impaired biofilm formation (**Figure 10A**). Interestingly, the biofilm formation of Δ*srf* mutant (FRY1) could not be restored by supplementing surfactin (**Figure 10A**). On solid MSgg medium, Δ*srf* mutants formed less structured and flattered colonies than the wild type after incubation for 2 or 4 days, while supplementing surfactin could partially rescue the colony architecture of Δ*srf* mutant (FRY1) (**Figure 10B**).

### Secretion of extracellular enzymes and solubilizing phosphates was decreased in Δ*srf* mutants of *B. amyloliquefaciens* Ba01

**Figure 11:**
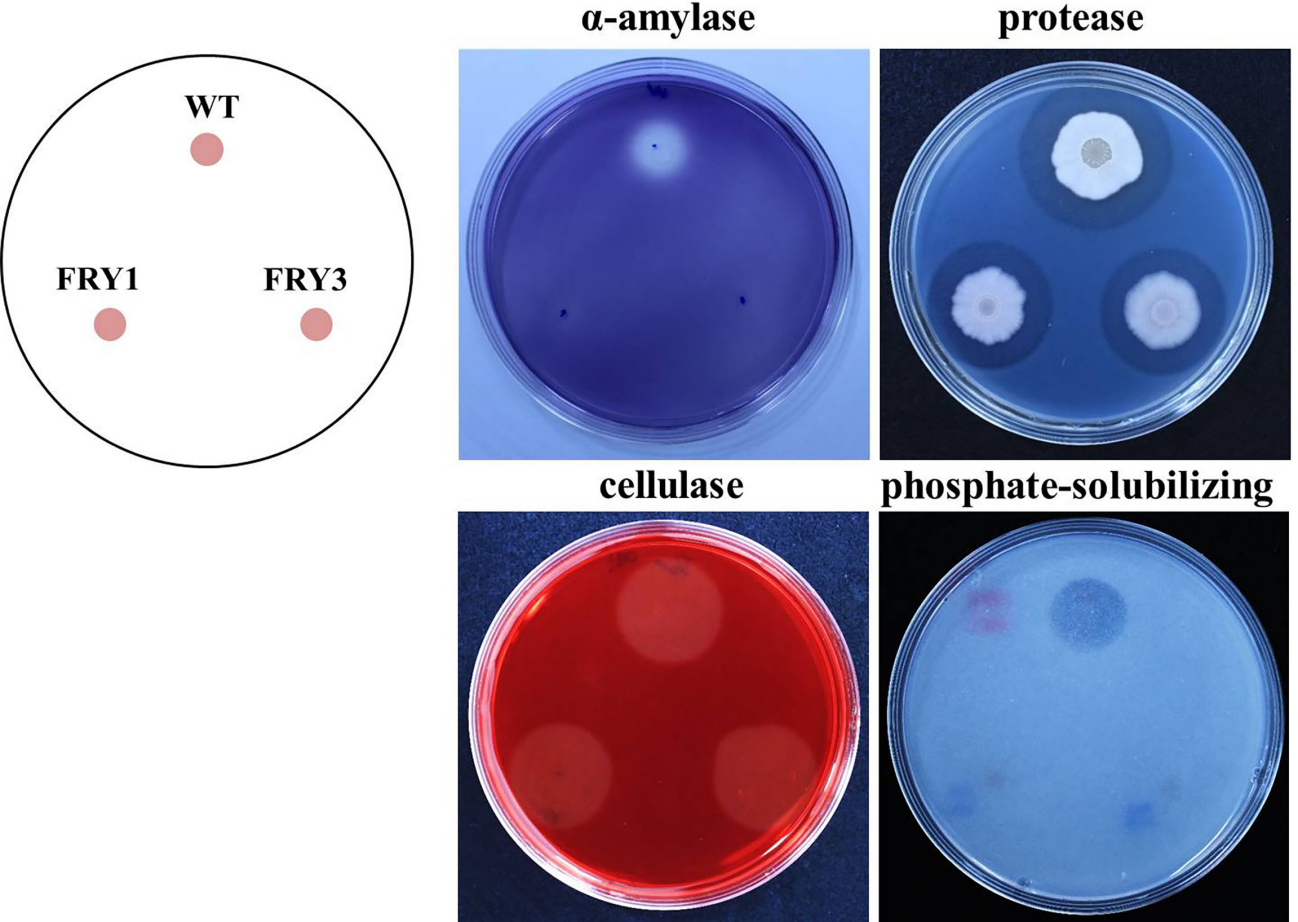
The enzyme activity of α-amylase and protease, and phosphate-solubilizing ability of Δ*srf* mutants were decreased. Three microliters of strains (OD_600_ = 1) were inoculated on media testing α-amylase, protease, cellulase and phosphate-solubilizing ability and incubated at 37°C for 2 days (α-amylase, protease, cellulase) or 5 days (phosphate-solubilizing).

In extracellular α-amylase activity and phosphate-solubilizing ability tests, we found that two Δ*srf* mutants could not grow normally and form clear zone in the same way as the wild type, indicating that α-amylase secretion and solubilizing phosphate activity was impaired in Δ*srf* mutants (**Figure 11**). In the protease test, although two Δ*srf* mutants could form clear zones [6.0 ± 0.16 mm (FRY1) and 5.5 ± 0.39 mm (FRY3)], they were significantly smaller than that of the wild type (7.4 ± 0.13 mm) based on *t*-test statistical analysis. However, in the cellulase test, wild type and Δ*srf* mutants grew normally and exhibited no difference in forming a clear zone (**Figure 11**).

### The Δ*srf m*utants could not inhibit growth and sporulation of *S. scabies* on potato tuber slices

**Figure 12:**
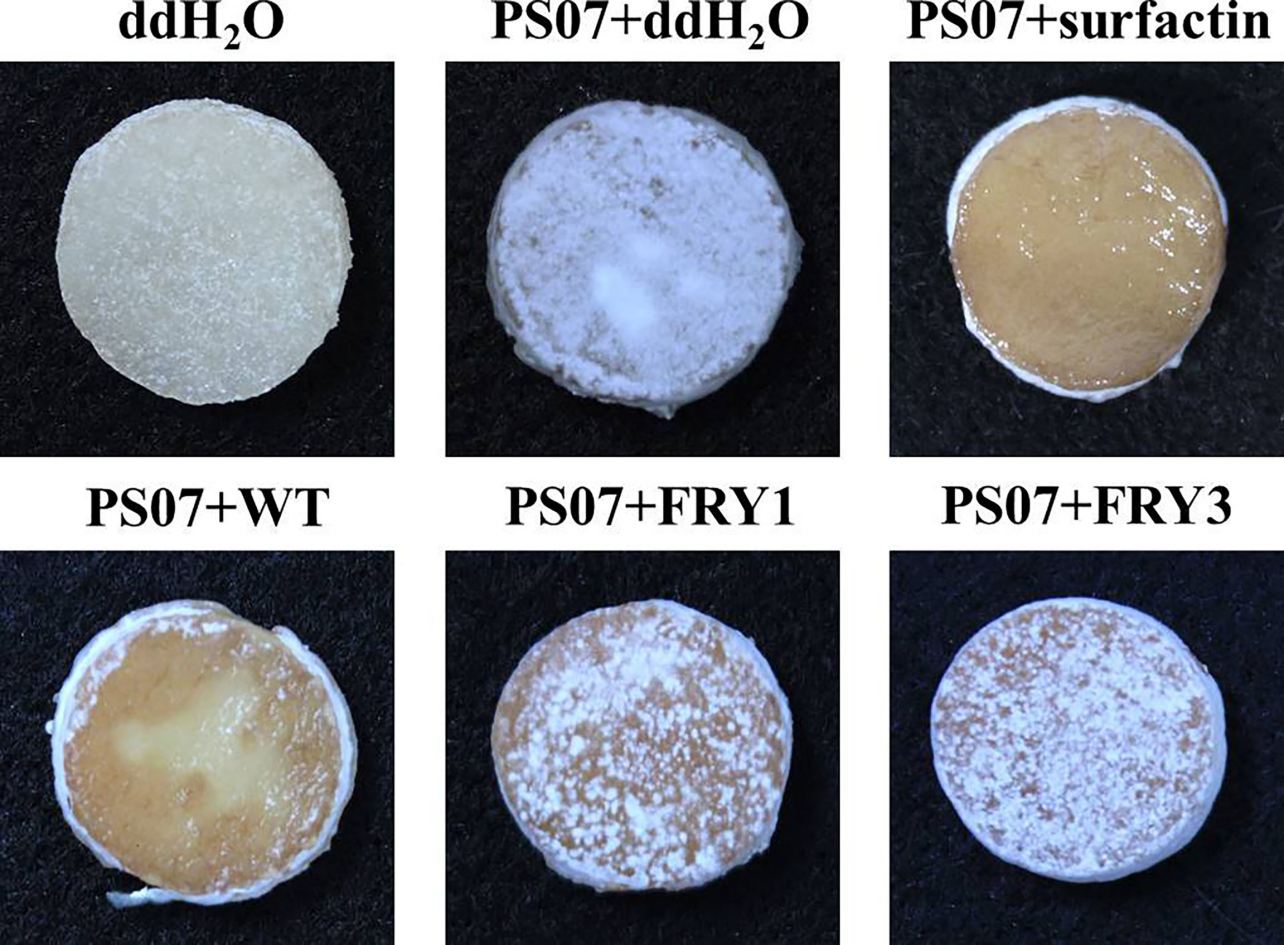
Two Δ*srf* mutants did not effectively inhibit the growth and sporulation of *S. scabies* PS07 on potato tuber slices. The 25 μl 10^8^ spores/ml of *S. scabies* PS07 spores were inoculated on potato tuber slices, incubated at 28°C for 1 day in the dark, and then 20 μl of Ba01 WT, FRY1, FRY3 (OD_600_ = 1), ddH_2_O or surfactin (0.1 mg/ml) were added to potato tuber slices. The samples were further incubated in the moist chamber at 28°C for 5 days before photographing.

In potato tuber slice assays, tuber slices treated with *S. scabies* PS07 were covered with *S. scabies* colonies and white spores; however, supplementing surfactin or adding *B. amyloliquefaciens* Ba01 effectively inhibited the growth and sporulation of *S. scabies* PS07 on potato tuber slices after incubation for 5 days at 28°C (**Figure 12**). In contrast, the two Δ*srf* mutants (FRY1 and FRY3) could not effectively inhibit the growth and sporulation of *S. scabies* PS07 on potato tuber slices (**Figure 12**).

## Discussion

Currently, many strategies have been developed to control potato common scab. Our previous study showed that *B. amyloliquefaciens* Ba01 can control common scab in the pot assay and field trial, but detailed mechanism remains unclear (Lin et al., 2018). In this study, we further demonstrated that the mechanism of Ba01 used to inhibit the growth of *S. scabies* is at least in part *via* secretion of surfactin since the deletion of Ba01 *srf* gene cluster resulted in reduced inhibition activity against *S. scabies*. The practical use of biocontrol agents to combat common scab is still present, although it is limited by various environmental or plant host factors. The environmental factors include temperature, humidity and soil pH/microbiota, etc., while host factors contain plant resistance to common scab causing pathogens. Besides biocontrol strategy, foliar application of auxin (2,4-dichlorophenoxyacetic acid) has been used to induce potato defense response against common scab disease and its mechanism is associated with enhanced tolerance to thaxtomin A (Tegg et al., 2008). This result demonstrates that auxin can ameliorate thaxtomin A toxicity. However, the role of surfactin secreted by *Bacillus* species on biosynthesis of thaxtomins is unclear, indicating a potential research topic in the future.

*Bacillus* species are important biocontrol agents in agriculture, and gene manipulation can be used as a tool to understand the mechanisms through which they combat plant diseases. However, there are relatively few studies using mutants of *B. amyloliquefaciens*, mainly due to its low transformation efficiency (Zhang et al., 2013; Zhang et al., 2015). *B. amyloliquefaciens*, which is a gram positive bacterium, has a thick cell wall that acts as a natural barrier when introducing plasmids into the cell. Moreover, *B. amyloliquefaciens* possesses a BamHI restriction-modification system, which prevents incorporation of exogenous DNA (Zhang et al., 2013; Zhang et al., 2015). In addition, whether plasmids can maintain and stably replicate also significantly influences the result of transformation. In comparison to domesticated strains, the strain used in this study, *B. amyloliquefaciens* Ba01, is a wild strain isolated from potato tuber, which makes it particularly difficult to transform. In this study, we tried three methods to carry out transformation, the first method was making competent cells according to Konkol team (Konkol et al., 2013); the second method was conjugation, using *E. coli* S17-1 as donor (Rachinger et al., 2013); and the third method was electroporation. Although we finally succeeded in transforming Ba01 and obtaining Δ*srf* mutants *via* electroporation, all three methods mentioned above had low transformation efficiency, with false positive transformants that did not result in plasmids growing on media containing antibiotics, or easy loss of plasmids during subculturing. Therefore, for future studies, an improved transformation method will be required to establish a stable method by which to study gene functions of Ba01.

Surfactin, which is a cyclic lipopeptide, has been found to be commonly secreted by several *Bacillus* species, and it plays an important role in *Bacillus* biology. According to previous studies, colonies of surfactin-deficient *Bacillus* mutants may become flat and unwrinkled, indicating that this molecule is required for normal colony morphology in some strains (Zeriouh et al., 2014; Luo et al., 2015; Zhang et al., 2022). Also, surfactin has been found to act as a signal molecule and triggers quorum sensing, which further facilitates multicellular behaviors such as swarming and biofilm formation (Shank and Kolter, 2011; Zeriouh et al., 2014; Luo et al., 2015). In this study, although addition of extracellular surfactin could restore the ability of Δ*srf* mutant to swarm and form normal colonies, biofilm formation was still impaired. Failure of additional surfactin to rescue biofilm formation was also found in *Bacillus subtilis* Δ*srfAA* mutant obtained by Luo team (Luo et al., 2015), while Zeriouh team discovered that the ability of *B. subtilis* Δ*srfAB* mutant to form biofilm could be restored by adding commercial surfactin (Zeriouh et al., 2014). This difference suggests the possibility that there might be some other proteins which regulate biofilm formation cooperatively with *srfAA* gene or other genes such as *srfAC* and *srfAD* in the *srf* gene cluster, and surfactin may play a role as a signal triggering the biofilm formation pathway.

Besides acting as a signal molecule, surfactin is also a strong biosurfactant, with the function of promoting moisture and reducing surface tension. In addition, according to our experimental results, surfactin is related to the sporulation ability of *B. amyloliquefaciens* Ba01, with Δ*srf* mutants having poor sporulation rate. Together with stimulation of biofilm formation, these functions facilitate swarming to nutrient rich niches and stable colonization, and enhance survival rate in the field, which are determinant factors that lead to efficient biocontrol capability of *Bacillus* species (Zeriouh et al., 2014; Aleti et al., 2016). In addition, there have been previous reports that indicated that surfactin is an ISR elicitor, which stimulates plant defense response in a wide range of crops (Cawoy et al., 2014; Chowdhury et al., 2015). This study also showed that surfactin may play an important role in plant-growth promotion, in which this compound seems to contribute to the enzyme activity of α-amylase and protease, and the phosphate-solubilizing ability of its producer. Consequently, we postulated that by producing surfactin, Ba01 could promote plant-growth and indirectly increase plant immunity to enhance the resistance of host plants against pathogens. All of the findings above suggest that surfactin production is essential for improving biocontrol ability of biocontrol microorganisms.

In conclusion, this study revealed that surfactin is a versatile compound. Besides its direct antagonistic activity against *S. scabies*, it is also related to swarming, biofilm formation, sporulation, phosphate solubilization and enzyme activity of α-amylase and protease. With these functions, *B. amyloliquefaciens* Ba01 is a potential candidate for controlling potato common scab. Combined with the findings of previous reports, there is also a chance that Ba01 could induce a systemic response in potato, providing another mechanism for combating potato common scab, which is worth further investigation. If surfactin does trigger induced systemic resistance in potato plants, besides using Ba01 as a biocontrol agent, direct application of surfactin may serve as an option as well, since the compound possesses pathogen growth inhibition and plant immunity enhancement ability simultaneously.

## Data availability statement

The original contributions presented in the study are included in the article/supplementary material. Further inquiries can be directed to the corresponding author.

## Author contributions

R-YF designed and conducted the experiments. Y-HC wrote the manuscript. CL, C-HT and Y-LY assisted in some experimental sections. Y-LC supervised the experiments and wrote the manuscript. All authors contributed to the article and approved the submitted version.

## Funding

This work is financially supported by grant NTU110L7811 from the Higher Education Sprout Project, Ministry of Education, and grants MOST 110-2923-B-002-002-MY3 and 110-2320-B-002-050-MY3 from the Ministry of Science and Technology, Taiwan.

## Acknowledgments

We are grateful to Miranda Loney for language editing, and Drs. Shih-Tung Liu and Wan-Ju Ke at Chang Gung University for providing *Bacillus* transformation protocol. This work is financially supported by grant NTU110L7811 from the Higher Education Sprout Project, Ministry of Education, and grants MOST 110-2923-B-002-002-MY3 and 110-2320-B-002-050-MY3 from the Ministry of Science and Technology, Taiwan.

## Conflict of interest

The authors declare that the research was conducted in the absence of any commercial or financial relationships that could be construed as a potential conflict of interest.

## Publisher’s note

All claims expressed in this article are solely those of the authors and do not necessarily represent those of their affiliated organizations, or those of the publisher, the editors and the reviewers. Any product that may be evaluated in this article, or claim that may be made by its manufacturer, is not guaranteed or endorsed by the publisher.
